# Physiotherapy Exercise Classification with Single-Camera Pose Detection and Machine Learning

**DOI:** 10.3390/s23010363

**Published:** 2022-12-29

**Authors:** Colin Arrowsmith, David Burns, Thomas Mak, Michael Hardisty, Cari Whyne

**Affiliations:** 1 Orthopaedic Biomechanics Lab, Holland Bone and Joint Program, Sunnybrook Research Institute, Toronto, ON M4N 3M5, Canada; 2 Halterix Corporation, Toronto, ON M5E 1L4, Canada; 3 Division of Orthopaedic Surgery, University of Toronto, Toronto, ON M5T 1P5, Canada; 4 Institute of Biomedical Engineering, University of Toronto, Toronto, ON M5S 3G9, Canada

**Keywords:** human activity recognition, pose detection, machine learning

## Abstract

Access to healthcare, including physiotherapy, is increasingly occurring through virtual formats. At-home adherence to physical therapy programs is often poor and few tools exist to objectively measure participation. The aim of this study was to develop and evaluate the potential for performing automatic, unsupervised video-based monitoring of at-home low-back and shoulder physiotherapy exercises using a mobile phone camera. Joint locations were extracted from the videos of healthy subjects performing low-back and shoulder physiotherapy exercises using an open source pose detection framework. A convolutional neural network was trained to classify physiotherapy exercises based on the segments of keypoint time series data. The model’s performance as a function of input keypoint combinations was studied in addition to its robustness to variation in the camera angle. The CNN model achieved optimal performance using a total of 12 pose estimation landmarks from the upper and lower body (low-back exercise classification: 0.995 ± 0.009; shoulder exercise classification: 0.963 ± 0.020). Training the CNN on a variety of angles was found to be effective in making the model robust to variations in video filming angle. This study demonstrates the feasibility of using a smartphone camera and a supervised machine learning model to effectively classify at-home physiotherapy participation and could provide a low-cost, scalable method for tracking adherence to physical therapy exercise programs in a variety of settings.

## 1. Introduction

Shoulder pain caused by symptomatic degenerative rotator cuff tears and low back pain (LBP) are highly prevalent conditions associated with decreased mobility and quality of life [1,2,3,4,5,6]. Conservative management with physical therapy has been established as an effective treatment leading to improved patient-reported outcomes for both of these conditions [7,8,9,10]. Essential to this effective management are high rates of patient participation in a physical therapy program [11,12]. Unfortunately, at-home participation in physiotherapy is often poor and decreases over time [11,12,13]. Current tools to measure adherence often rely on patient-reported diaries which are subject to low rates of completion and can suffer from a range of other biases [14,15]. Establishing objective measures of adherence is therefore a clinically useful component of physiotherapy and remains a challenging problem [15,16].

In recent years, wearable inertial measurement units (IMUs), such as those contained in widely available smartwatches and smartphones, have been used for a variety of human activity recognition tasks [17,18,19]. In the context of physical therapy, ref. [20] developed a smartwatch-based sensor system which was able to detect shoulder physiotherapy exercises using hand-crafted IMU time series features or a three-layer convolutional neural network (CNN) [12,21]. This sensor system was subsequently expanded by [22] to eight IMUs worn around the body, and it was shown that a system of three IMUs worn on the low back, thigh, and ankle could be used to classify low-back exercises. Although these systems have been shown to be effective in measuring physiotherapy performance, they require hardware configurations that are highly-specific to the exercise type (watch vs. pants) and required substantial development to expand the approach from the shoulder exercises to those performed for other anatomical sites (i.e., the lower back) [20,22].

Video data offer the potential ability to measure the movement of the entire body. Existing platforms often rely on specialized hardware (e.g., Microsoft Kinect [23]) or costly motion capture systems [24]. In particular, support vector machines (SVM) have been trained on keypoints obtained from Microsoft Kinect systems for emotion and gesture recognition [25]. Pre-trained image classification models have also been used to extract features from video frames, with an SVM used to classify postural control metrics [26]. Ref. [27] used a custom configuration of off-the shelf IMUs coupled with a depth camera and hand-crafted algorithms to compute gait and posture metrics. However, these methods lack the ability to run directly on a smartphone without any additional customized hardware. Recently, the emergence of open source pose detection frameworks such as OpenPose [28], MoveNet [29], and BlazePose [30] has made direct biomechanical analysis possible with single-camera videos. Machine learning models have been trained with 2D pose keypoints for predicting gait metrics such as walking speed and cadence [31,32] and fall detection [33]. The increased availability of open source pose detection models capable of running in real-time on most smart phones (or other consumer electronics containing cameras such as smart-home devices) offer the potential to provide a scalable platform for the detection of a wide variety of physical therapy exercises with a single camera. However, to our knowledge, pose detection and time series models have not been used to directly classify physiotherapy activity.

The purpose of this study was to evaluate the suitability of using a single camera to detect and classify physiotherapy exercises in a variety of anatomic locations. To test this, we developed and optimized a proof-of-concept system for classifying the videos of exercises from both a shoulder and an LBP physical therapy program with machine learning. In addition, we performed an analysis of the model’s robustness to variation in the camera angle and assessed the minimum number of subjects required to train such a model. It is hypothesized that videos of shoulder and low back physiotherapy exercises could be classified based on the temporal changes in the keypoint locations estimated by a pose detection model. Furthermore, we hypothesize that model architectures used to classify the temporal signals of the inertial data of physiotherapy exercises could be used to classify keypoint time series derived from video data. Although we apply these models to physiotherapy exercise classification, they could provide a platform for scalable activity recognition in a wide range of applications such as physical rehabilitation, remote care, gait analysis, and sports and fitness.

## 2. Materials and Methods

### 2.1. Dataset

Exercises from two evidence-based rehabilitation protocols were used in this study. Seven exercises used to treat full-thickness atraumatic rotator cuff tears [7] were chosen for the shoulder activity task. Seven exercises used by [22] from the McKenzie low-back physiotherapy framework [34] were selected for the low-back exercise task. Both sets of exercises were chosen to incorporate movement in a variety of planes which are typical of exercises prescribed for rotator cuff tears and LBP, respectively. The full list of exercises performed for each task can be found in Appendix A. Exercises are referred to as “symmetrical” if the movement was bilateral, with both sides of the body moving in unison (e.g., push-ups). “Asymmetrical” exercises refer to unilateral movements which are performed to one side (e.g., internal rotation with the left arm).

Twenty-one healthy adult subjects with no prior history of low-back pain or shoulder rotator cuff pathology were recruited for this study. Subjects provided informed consent to participate in a study approved by the Sunnybrook Health Sciences Centre Research Ethics Board (REB # 3505).

Participants performed 10 repetitions of each exercise from both the shoulder and low-back tasks while being filmed with two smartphone cameras. Asymmetrical exercises were performed for five repetitions on each side. Because each exercise is performed with a slightly different body position and orientation, the camera positioning relative to the participant was specific to each exercise. The two cameras were positioned at an angle of 45${}^{\circ }$–90${}^{\circ }$ apart relative to the participant, with as much of the participant’s body in view as possible. One camera angle per activity class was used for model optimization and experimentation. The recordings from the second camera angle for each exercise were held out in order to assess the model’s robustness to camera angle. Participants were filmed in a variety of settings which incorporated various types of lighting, backgrounds and occlusion. The exercise type, participant number, camera angle, and body side (e.g., left, right, or symmetrical) were labelled by researchers for all videos.

**Figure 1:**
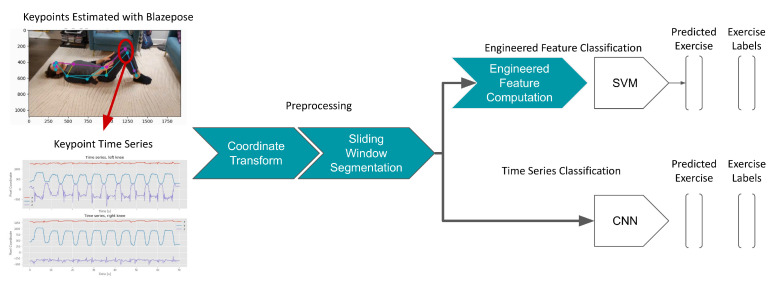
The design of the proposed platform. Subjects are filmed with a single camera using a smartphone while performing physiotherapy exercises. Joint keypoints are estimated for each video frame using BlazePose, resulting in a timeseries of keypoint coordinates for each video. A coordinate transform is applied to the keypoint timeseries in addition to sliding window segmentation. The keypoint time series segments are then used to train and evaluate a convolutional neural network (CNN). As a baseline comparison, engineered features are computed for each time series segment and used to train a support vector machine (SVM). Both models are trained to predict the physiotherapy exercise being performed in the given segment (seven-class classification). This process was performed for shoulder activities (Table A1) and again for low-back activities (Table A2).

The data processing and modeling methodology is summarized in Figure 1. Thirty-three-keypoint skeletons were extracted for all frames of all single-camera videos using BlazePose [30]. BlazePose was implemented using the MediaPipe Python package [35] and was found to run with an average frame rate of 32 frames per second on an Intel i5 CPU. Each keypoint was represented by a four-axis vector containing the *x*, *y*, *z*, image coordinates of the keypoint in addition to the “visibility” *v* of the keypoint. The resulting datasets for low back $D_{LB}=\left\{\left(X_{i},y_{i}\right){|}_{i=0}^{N}\right\}$ and shoulder $D_{SH}=\left\{\left(X_{i},y_{i}\right){|}_{i=0}^{M}\right\}$ containing time series (derived from *N* and *M* videos, respectively) and ground truth exercise labels $y_{i}$ were used for classification model training. Each time series $X_{i}$ was represented by a matrix of keypoints $k_{i}$ containing the four-axis keypoint coordinates for *n* frames:

(1) $$\begin{array}{c} X_{i}=\left\{K_{1},K_{2},\ldots ,K_{33}\right\} \end{array}$$

(2) $$\begin{array}{c} K_{i}=\left\{x,y,z,v\right\}. \end{array}$$

The matrices $X_{i}$ were subsequently flattened to shape (132, *n*) for model training so that for the *j*th frame in the *i*th video, we have:

(3) $$\begin{array}{c} X_{i,j}=\left(k_{1x},k_{1y},k_{1z},\cdots ,k_{33v}\right). \end{array}$$

### 2.2. Preprocessing

All sequences were resampled to a sampling rate of 25 Hz using cubic interpolation. Resampled values are computed by fitting a third-order spline to the data and interpolating new values at the specified sampling rate. Each resampled skeleton time series was segmented using a sliding window segmentation with a window width of 400 samples (16 s). This sampling rate and window width were chosen via a grid search, with a limit of 20 s (roughly two repetitions) placed on the possible window width. A window stride of 50 samples (2 s) was used as a data augmentation strategy. Using a smaller stride value effectively creates increasingly overlapping windows, thus increasing the size of the dataset. All interpolation and segmentation steps were performed using Seglearn, an open source Python package [36].

### 2.3. Exercise Classification Models

Two time series classification models were used in this study. First, a support vector machine (SVM) classifier, trained on hand-crafted time series features, was used as a baseline model. Eleven engineered features were computed for each segmented keypoint time series using the Seglearn Python package [36]. The resulting features were normalized to zero mean and unit variance and used to train a SVM model with a linear kernel and a regularization parameter of 0.025. Feature normalization and model training was performed using the Scikit-Learn Python package [37]. The SVM was chosen as the baseline model due to its relative simplicity and interpretability as a classifier.

A convolutional neural network (CNN) was also trained directly on keypoint time series segments. The CNN architecture proposed by [38] was adopted for this study. This model architecture was chosen because it is considered a strong baseline for time series classification [39] and has been found to be effective in activity classification tasks with IMU data [22,38]. This relatively simple CNN architecture has been shown to outperform models with more modern architectural features such as skip connections or LSTM layers in time series classification tasks [39]. The implementation of this CNN in this study consisted of three 1D convolutional layers, each with 128, 256 and 128 feature maps, respectively. Each convolutional layer was followed by batch normalization and a rectified linear unit (ReLU). Global average pooling was used after the last convolutional layer. This improves the model’s robustness to temporal translations and has been shown to lead to optimal performance in inertial classification tasks [39]. After global average pooling, $L^{2}$ normalization was performed, followed by a fully connected layer with softmax activation. The CNN was trained using the Adam optimizer with categorical cross entropy loss for 50 epochs and a learning rate of 0.005. Softmax activation and the Adam optimizer are both widely used for optimizing CNNs for classification [40,41] and were chosen for their success in previous classification tasks with IMU time series [20,22,38]. All CNN models tested in this study had identical architectures with the exception of different numbers of input channels due to the keypoint combinations as described in Section 2.6.1.

### 2.4. Baseline Model Optimization

A grid search was employed to optimize the keypoint combinations, input channels, coordinate transforms and window width, in addition to model-specific hyperparameters for both the SVM and CNN models in each classification task. The search included the {x,y}, {x,y,z} and {x,y,z,v} input channel combinations along with the keypoint combinations and coordinate transforms described in Section 2.6.1 and Section 2.6.2. Window widths of 50, 100, 200, 400, and 500 samples were tested. The learning rate of the CNN was tuned, with values of 0.01, 0.005, 0.001, and 0.0001 were tested. The optimized model settings for each classification task are shown in Table 1. The CNN model for each classification task was used for subsequent experiments. All optimized models used a window width of 16 s and a sampling rate of 25 Hz.

### 2.5. Performance Evaluation

All experiments were trained and evaluated using a 5-fold cross validation approach, splitting folds by participant. This ensured that recordings from the same patient did not appear in both the training and test sets. The same splitting strategy was used for each experiment, ensuring that the records contained in each fold were consistent throughout our study. The mean class-balanced accuracy and 95% confidence interval across folds are reported for each experiment.

### 2.6. Experiments

#### 2.6.1. Keypoint Combinations

The BlazePose pose detection model returns a skeleton of 33 body keypoints for each frame. However, not all of these keypoints may be required for effective activity classification. The performance of the model in classifying activity when trained on a variety of BlazePose keypoint combinations was therefore assessed. Five keypoint combinations were selected based on their relevance to the biomechanics of the physiotherapy activities and in consideration of standard keypoint sets used in other pose detection frameworks. Each set described here is a subset of the pose keypoints returned by BlazePose:

- **All Keypoints**: The full set of 33 BlazePose keypoints.
- **All Without Face**: Twenty-two keypoints containing the BlazePose set without keypoints on the face.
- **COCO Keypoints**: Set of 17 keypoints used in the COCO [42] dataset. These are a subset of the BlazePose set which contain fewer keypoints on the face and hands.
- **Major joints**: Twelve keypoints made up of the shoulders, elbows, wrists, hips, knees and ankles.
- **Upper Body Joints**: Eight keypoints made up of the shoulders, elbows, wrists, and hips.

The effect of each keypoint set on model performance was evaluated for the CNN using 5-fold cross validation, splitting folds by participant. Only keypoint time series from videos filmed from one camera angle per exercise were used in this experiment.

#### 2.6.2. Coordinate Transforms

Two coordinate transforms were developed in order to account for the participant’s position and orientation in the image field of view. A translation was applied to the skeletons by computing the point midway between the hips (BlazePose keypoints 24 and 25) and setting this as the origin $K_{o}$, resulting in the translated set of keypoints $X_{i}^{t}$ for the *i*th record

(4) $$\begin{array}{c} K_{o}=\frac{K_{24}+K_{25}}{2} \end{array}$$

(5) $$\begin{array}{c} X_{i}^{t}=X_{i}-K_{o}. \end{array}$$

A rotation transformation was also developed order to account for the orientation of the participant’s body relative to the camera. In each frame, a new set of orthonormal basis vectors $\left\{\hat{x},\hat{y},\hat{z}\right\}$ were computed such that $\hat{x}$ and $\hat{y}$ are in the plane formed by the shoulders and $K_{o}$. These basis vectors are then stacked to create the rotation matrix R which is applied to all keypoints in the frame, resulting in the translated and rotated keypoints $X_{i,j}^{t}$ for the *j*th frame in the *i*th record:

(6) $$\begin{array}{c} R=\{\hat{x},\hat{y},\hat{z}\} \end{array}$$

(7) $$\begin{array}{c} X_{i,j}^{r}=RX_{i,j}^{t}. \end{array}$$

In order to assess the impact of transforms on model performance, three CNN models were trained and validated using 5-fold cross validation, with each model using one of the following transforms during preprocessing:

- **None**: No transform was applied. The raw keypoints in image pixel coordinates from BlazePose were passed to the CNN.
- **Translation**: The translation transformation was applied to all keypoint timeseries.
- **Translation and rotation**: The translation followed by a rotation was applied to all keypoint timeseries.

Only records from one camera angle per exercise were used in this experiment. The “visibility” of each keypoint was left unchanged during translation and rotation transformations.

#### 2.6.3. Camera Angles

Robustness to different camera angles is essential for the effective deployment of a video-based classification system. As such, the CNN’s classification performance on recordings filmed from previously unseen camera angles in our dataset was evaluated. Additionally, the effects of coordinate transforms (Section 2.6.2) on model performance for those angles was investigated. This experiment was performed in two stages: First the three CNN models were trained on records from only one camera angle per exercise, with each model using one of the transforms from Section 2.6.2 (none, translation, translation followed by rotation). The three models were then tested on records from the first *and* the second camera angle for each exercise. In the second stage of this experiment, the three CNN models were trained on both the first and second angle for each exercise, and tested on both the first and second angle. Both stages of the experiment employed the same 5-fold cross validation, splitting folds by subject to avoid data leakage. Results are reported as the mean ± 95% CI across the five folds.

#### 2.6.4. Training Saturation

The validation performance of the CNN with respect to the amount of data used to train the model was also assessed. The same subject-split 5-fold cross validation used in experiments Section 2.6.1, Section 2.6.2 and Section 2.6.3 was created. A random subset of subjects in the training set of each fold was used to train the CNN and the validation set was used to test the model. This was repeated for a variety of training set sizes, each time testing on the same held-out validation set. This was performed using only records filmed from one camera angle for each exercise for both the low-back and shoulder classification tasks. Results are reported as the mean accuracy across the five folds, ± the 95% confidence interval.

## 3. Results

### 3.1. Baseline Models

The SVM and CNN models were optimized for each classification task. Models and input settings were optimized with a grid search across model hyperparameters, keypoint combinations, channels (*x*, *y*, *z*, *v*), and coordinate transforms using only one camera angle for each exercise. The optimized keypoint parameters and the resulting performance in 5-fold cross validation are reported in Table 1. Although the highest classification accuracy was achieved by the CNN model for low back exercise classification (0.995 ± 0.009) and the SVM provided the best performance for shoulder exercise classification (0.972 ± 0.016), neither model significantly outperformed the other within each classification task. The runtime of the preprocessing and classification pipeline was 0.28 ± 0.07 s (mean ± SD) for each record on an Nvidia Titan RTX 24GB GPU, with the mean record length of 66 ± 20 s (mean ± SD). The optimized CNN models for both classification tasks were used for subsequent experiments.

### 3.2. Keypoint Combinations

**Figure 2:**
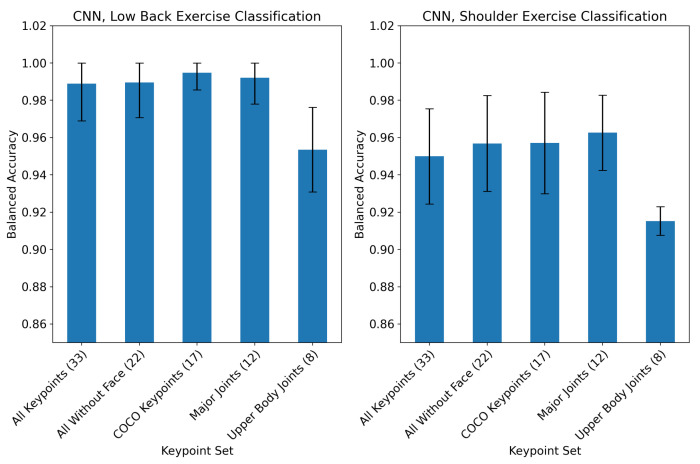
The effect of pose keypoint combinations on CNN classification performance for the low back (**left**) and shoulder (**right**) exercise classification tasks. The mean ± 95% CI class-balanced accuracy across 5-fold cross validation is shown. All models were trained and validated using only one camera angle for each exercise.

The effect of keypoint selection on CNN exercise classification performance for the both the low back and shoulder tasks is plotted in Figure 2. The model performance degraded significantly for the “upper body joints” keypoint set in both classification tasks. All other keypoint combinations that were tested resulted in at least 98% accuracy for low back and at least 94% accuracy for shoulder exercise classification.

### 3.3. Coordinate Transforms

**Figure 3:**
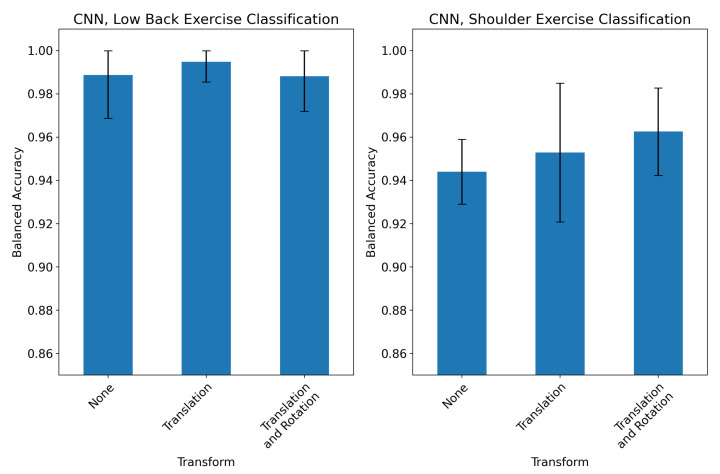
Effect of coordinate transforms on classification model performance for low back (**left**) and shoulder (**right**) exercises, using only videos from one camera angle for each exercise. Plots show the mean ± 95% CI class-balanced accuracy in a 5-fold cross validation experiment, creating folds by participant. The transform (either no transform, translation, or translation and rotation) was applied to both training and validation records in each fold.

The impact of applying a translation and/or rotation to BlazePose keypoints prior to CNN training is assessed in Figure 3. Model performance on low-back classification was not significantly affected by either transform, although a translation and rotation resulted in an increased inter-fold variability. Each additional transform did offer a slight improvement in performance in the shoulder exercise classification task, although both models which used transforms were within the 95% confidence interval of the model trained on raw keypoints.

### 3.4. Camera Angles

**Figure 4:**
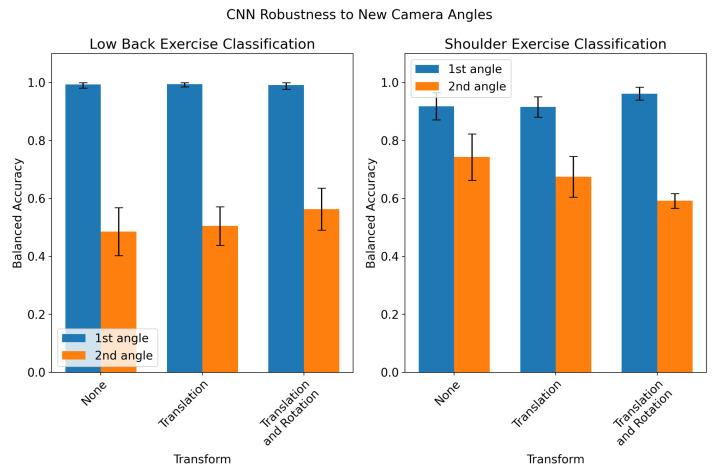
CNN robustness to new camera angles. The CNN was trained on records filmed from one camera angle (“1st angle”, in blue) and tested on a held-out set of records filmed from the second camera angle for each exercise (“2nd angle”, in orange). This was performed using a 5-fold cross-validation approach, splitting the folds by subject to prevent data leakage. This experiment was repeated for low-back exercise classification (**left**) and shoulder exercise classification (**right**), and evaluated the impact of coordinate transforms (no transform, translation, or translation and rotation).

**Figure 5:**
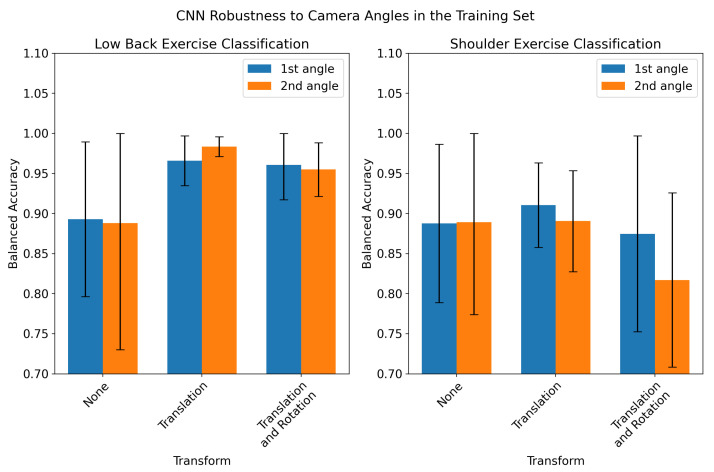
CNN robustness to camera angles in the training set. The CNN was trained on records from both camera angles for each exercise and tested on both camera angles in a 5-fold cross validation approach, splitting folds by subject. This was repeated for both low back (**left**) and shoulder (**right**) classification tasks, using each coordinate transform.

The performance of the CNN in classifying exercises recorded for a previously unseen camera angle is shown in Figure 4. The model performance is significantly degraded when classifying records from the second angle (up to 50% decrease in accuracy). Coordinate transforms did not have a significant effect on performance in the low back exercise classification task and resulted in degraded performance in the second angle for shoulder exercise classification. The performance of the CNN when trained with records collected from both angles is shown in Figure 5. When trained on records filmed from both the first and second angle, the CNN’s performance was not significantly different for the two angles, although the inter-fold variation in accuracy was much higher than the baseline models in Table 1. However, when training on both angles, the CNN performance on the two angles was lower than the baseline models by 2–5%. Additionally, the coordinate transforms provided a 7–10% increase in accuracy for low-back exercise classification when training and testing on both angles.

### 3.5. Training Saturation

**Figure 6:**
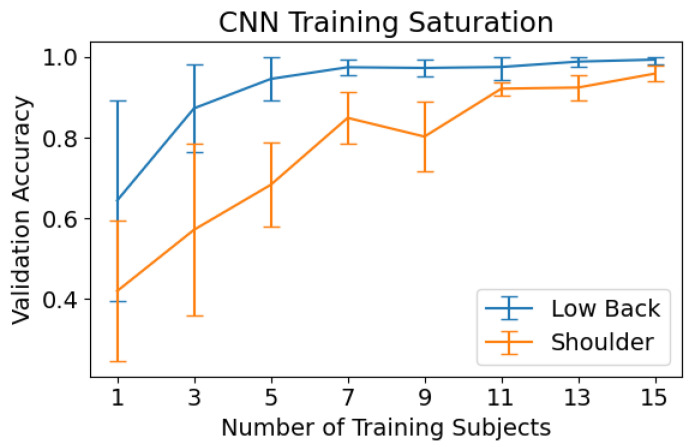
CNN performance as a function of training set size. The CNN was trained on a random subset of subjects from the training set of a 5-fold cross validation split and tested on a constant held-out validation set. The mean accuracy ± 95% CI is shown for various training set sizes (displayed as the number of subjects). This is shown for both the low back (blue) and shoulder (orange) classification tasks. Only records from one camera angle for each exercise were used in this experiment.

The performance of the CNN with respect to the training set size is shown in Figure 6. The CNN’s performance on low back exercise classification degraded significantly when trained on fewer than seven subjects. When training on seven or more subjects, the CNN achieves near optimal classification accuracy. The model does not reach this same plateau in performance for shoulder classification. An increase in shoulder activity classification performance is shown as the number of training subjects increases, with this increase slowing above eleven subjects.

## 4. Discussion

This work describes the development and evaluation of machine learning models (SVM and CNN) for classifying videos of low-back and shoulder physiotherapy activities based on time series data derived from pose detection keypoints over the course of the video. The CNN achieved a performance on par with the SVM baseline when trained and evaluated on time series derived from the videos of a single camera angle for each exercise. All models achieved a classification accuracy above 95% in both low-back and shoulder exercise classification tasks. Models performed better on the low back task, perhaps due to the wider variety of full-body movements involved in those exercises. The low-back protocol included two exercises which were performed while standing, and several of which were performed while lying on the ground in various configurations.

Further investigation into the use of different possible keypoint combinations as inputs to the model showed that any keypoint combination which includes at least the major joints (shoulders, elbows, wrists, hips, knees and ankles) resulted in optimal performance. As a result, a wide variety of pose detection models which offer various keypoint topologies such as BlazePose (33 keypoints), MoveNet (17 keypoints), or OpenPose (15, 18, or 25 keypoints) could offer viable inputs to a CNN. This improves the flexibility of our model and increases the number of options available for cross-platform deployment in a home setting.

When deploying a video-based classification system into a home setting, it is crucial that the system is invariant to variation in the camera setup. In particular, the location of a subject in the camera frame and the orientation of the subject relative to the camera must be accounted for. In order to do this, we leverage the relatively recent emergence of 3-dimensional, single-camera pose detection models. BlazePose produces a three-dimensional representation of joint locations for each video frame. The *x* and *y* values of these 3D joint locations are represented as pixel coordinates within the frame and the *z* values reflect an estimate of the “depth” of the joint in or out of the frame. These keypoint locations are therefore highly dependent on the position of the participant in the camera frame, as well as the orientation of the participant (e.g., facing the camera, back to the camera, side-on to the camera, etc.). In order to study the effects of these parameters, camera angles relative to each participant were labelled and the dataset was separated into two subsets: one set of records which used the first camera angle for each exercise, and one set which used the second camera angle for each exercise. We then used these two datasets to study the robustness of our models to different camera angles.

**Figure A1:**
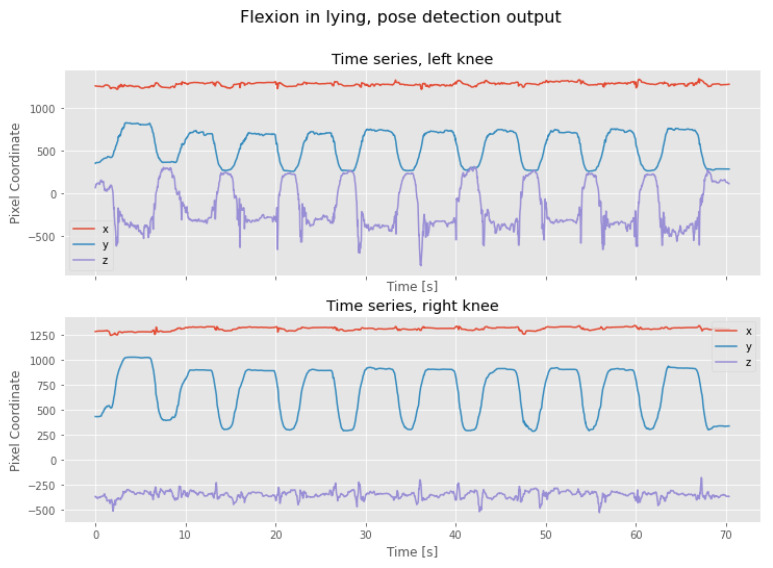
Sample keypoint time series from one video of a subject performing flexion in lying. The top plot shows the *x*, *y* and *z* coordinates of the left knee over the course of the video, estimated by BlazePose. The right knee is shown in the bottom plot.

We also developed two coordinate transforms which attempted to account for the location and orientation of the participant in the camera frame. Neither the translation nor rotation resulted in significant improvement in CNN performance when training and testing on the first angle from each exercise (Figure 3). We also evaluated the CNN’s robustness to new camera angles by testing the model on held-out records from the second camera angle (Figure 4). The model performed significantly worse on records from the held-out angles when no transformation was applied to input keypoints. This was expected, since in the absence of a coordinate transform, videos taken from different angles would create different keypoint time series. However, applying a translation and/or rotation to the training and held-out records did not significantly improve performance. For shoulder exercise classification, the transforms resulted in a slight decrease in CNN performance on held-out records. The ineffectiveness of these transforms to account for camera angles may be due to the high variance of the *z* axis in 3D pose-detection models. The visual inspection of the keypoint time series revealed that the *z* channel contains significantly more high-frequency noise than the *x* and *y* channels. An example of this is shown in Figure A1. It is likely that the rotation described in Equation (7) propagates this noise across all three dimensions of the rotated signal. In order to test whether we could train the model itself to be robust to multiple angles, we retrained the CNN on records collected from both angles, shown in Figure 5. The resulting model had virtually equal classification performance on records filmed from either angle. However, the translation and rotation transforms did offer improved performance in low-back classification for both angles compared to using no coordinate transform. Additionally, the performance of the CNNs when trained on records from multiple angles was lower than the models trained and tested on a single angle by only 2–5%. Our results suggest that training the CNN on multiple angles coupled with a translation transformation is an effective way of making a model robust to variations in the camera angle which occur in a home environment.

Results across all our experiments showed that the model classification of low-back physiotherapy exercises was consistently better than shoulder exercise classification. This could be explained by the different characteristics of the exercises in the two datasets. In particular, the shoulder dataset contained more asymmetrical exercises. This would result in roughly half the amount of training data for these exercises, since only five repetitions were performed for each side, compared to ten for symmetrical exercises. Furthermore, only records from one camera angle-side combination were used for each exercise. This theory is supported by the training saturation results in Figure 6 which show that, unlike in low-back classification, the CNN does not reach a plateau in performance for shoulder exercise classification as more training subjects are used. This suggests that it is likely that adding more training data could improve the performance of the model on shoulder exercise classification.

Although the effect of the camera angle on model performance was explored in quantitative experiments, this study was limited by the availability of only two camera angles for each exercise. Prior to deployment into a clinic or a home setting, models would have to be retrained on a wide range of possible camera angles. Unfortunately, the lack of publicly available datasets of videos of physiotherapy exercises with labelled camera angles makes this difficult. Additionally, this study only included healthy participants. An investigation of the CNN’s ability to generalize to patients with low back or shoulder pathology performing these exercises is crucial to the successful deployment of this system. A further limitation of our study was the use of only the SVM and CNN models. Testing a wider selection of engineered feature models (k-nearest neighbours, random forest, XGBoost, etc.) may have yielded a higher performance. Alfakir et al. [22] compared the performance of nine engineered feature models in classifying IMU time series of low-back physiotherapy and found that XGBoost and random forest models performed best. However, rather than performing an exhaustive search of model candidates, the purpose of our study was to test the ability of a CNN architecture optimised for IMU classification to generalize to video keypoint time series classification. To provide a baseline for comparison, we chose one engineered feature model (the SVM) due to its simplicity and interpretability.

Recently, several studies have used video-based pose detection models to estimate a range of biomechanical metrics. In particular, single-camera pose keypoints have been used to directly compute various temporal gait parameters [43]. In an approach similar to ours, several studies have trained time series machine learning models on single-camera pose keypoint data to predict gait parameters such as walking speed [31,32]. Other studies have used the transfer learning of pre-trained image classification models to classify videos of upper-limb tension tests on a single-frame basis [44]. Ref. [45] used dynamic time warping to compare the pose keypoint time series of a patient and a coach and derive an exercise performance score of lower limb exercises. However, to our knowledge this is the first study to directly classify the entire recordings of physical therapy exercises. Classification provides a direct, actionable outcome which can be used to track adherence, whereas predicting biomechanical movement metrics requires another layer of modeling or interpretation in order to derive actionable outcomes. To our knowledge, this is also the first study to directly study the dependence of the camera angle on the performance of classification models. Ref. [46] avoided the issue of camera angle dependence by combining the 2D pose estimates from two cameras into a single 3D keypoint representation. However, this would face significant deployment barriers in a home setting (requiring two cameras recording simultaneously and perhaps consistent positioning). Using two or more cameras would require the user to position both at specific angles and ensure they are in the frame of view of both cameras at all times. Our proposed solution of training models on records collected from multiple camera angles is designed to allow a home-based application to operate effectively and robustly on data acquired from a single smartphone camera.

The CNN architecture chosen in this study was originally optimised to classify the IMU time series of physiotherapy activities [38]. When trained on the signals of pose keypoints, this model proved to be an effective classifier of videos of physiotherapy activities. Minimal effort was required to extend the model from shoulder activity classification to low-back activity classification. In contrast, when expanding an IMU-based model to a new anatomic location or activity type, significant optimization of the hardware setup and sensor locations is required [22]. This suggests that a future video-based application for at-home physiotherapy participation measurement could be scaled to a wide range of activities with limited development effort required.

Future directions for this work should include testing these models on patients undergoing low-back or shoulder physiotherapy in a home setting. Prior to this, it would be crucial to retrain the CNN on keypoints collected from a wider variety of camera angles in order to ensure that the system is robust to variation in the camera angle. Additionally, it is anticipated that factors such as poor lighting and occlusion are more likely to occur in an uncontrolled home setting and could hinder keypoint extraction. Thus, the robustness of both the keypoint detection and the CNN classifier should be studied with respect to environmental factors. Finally, further deployment of this system into a remote care setting would require the development of a smartphone application to extract the pose keypoints and run the classification CNN.

## 5. Conclusions

Classification models trained on the time series of keypoints from pre-trained pose detection models can effectively classify the videos of physiotherapy exercises. Furthermore, this technology can be easily extended to multiple anatomical sites and exercise types. These models can learn to account for videos filmed from multiple camera angles with very little loss in classification accuracy. Finally, datasets for model training can be created with as few as seven to eleven participants. However, this study was performed on healthy participants. This technology should be tested on patients undergoing shoulder or low-back physiotherapy in a home setting prior to widespread deployment. The ability to use single-camera videos to measure physiotherapy activity lowers the bar to entry for many users and removes the requirement for specialized hardware. This proof-of-concept work is an important step towards developing a scalable application for measuring physiotherapy adherence in a home setting.

## Figures and Tables

**Table 1:** The optimized models used for subsequent experiments. For each model (CNN and SVM) and each classification task (low back and shoulder), a grid search was performed to select the optimal input channels, keypoint set, and coordinate transforms. All models used a window width of 16 s and a sampling rate of 25 Hz. Model accuracies in a 5-fold cross validation experiment, split by subject, using videos from only one camera angle per exercise are shown below.

Model	Classification Task	Channels	Keypoints	Transforms	Accuracy
SVM	Low back	{x,y}	Major joints	Translation	0.992 ± 0.011
SVM	Shoulder	{x,y,z,v}	BlazePose without face	None	0.972 ± 0.016
CNN	Low back	{x,y,z,v}	COCO	Translation	0.995 ± 0.009
CNN	Shoulder	{x,y,z,v}	Major joints	Translation and rotation	0.963 ± 0.020

## Data Availability

Seglearn Python package used for preprocessing in this study can be found here https://github.com/dmbee/seglearn, accessed on 4 November 2021. The Keras code used to create the CNN model is available https://github.com/dmbee/fcn-core, accessed on 4 November 2021.

## Abbreviations

The following abbreviations are used in this manuscript:

SVM	Support vector machine
CNN	Convolutional neural network
LBP	Low-back pain
IMU	Inertial measurement unit

## Appendix A. List of Low-Back and Shoulder Physiotherapy Exercises

**Table A1:** Shoulder physiotherapy exercises used in the study.

Exercise Name	Symmetrical
Abduction stretching	Yes
Flexion	No
Wall push-ups	Yes
External rotation	No
Internal rotation	No
Row	Yes
Pull downs	Yes

**Table A2:** Low-back physiotherapy exercises used in the study.

Exercise Name	Symmetrical
Sustained prone position	Yes
Dynamic extension in standing	Yes
Dynamic extension in lying	Yes
Dynamic flexion in lying	Yes
Flexion rotation with one leg	No
Flexion rotation with both legs	No
Dynamic side glide in standing	No
